# Th1 cytokines in pediatric acute lymphoblastic leukemia

**DOI:** 10.1007/s00262-023-03512-5

**Published:** 2023-08-23

**Authors:** Sarah Schober, Jennifer M. Rottenberger, Johannes Hilz, Evi Schmid, Martin Ebinger, Tobias Feuchtinger, Rupert Handgretinger, Peter Lang, Manon Queudeville

**Affiliations:** 1 Department I – General Pediatrics, Hematology/Oncology, University Children’s Hospital, Eberhard Karls University Tuebingen, Tuebingen, Germany; 2 Department of Pediatric Surgery and Pediatric Urology, University Children’s Hospital, Eberhard Karls University Tuebingen, Tuebingen, Germany; 3 Dr. Von Hauner University Children’s Hospital, LMU, Munich, Germany; 4 Division for Pediatric Stem Cell Transplantation and Immunology, Clinic for Pediatric Hematology and Oncology, University Medical Center Hamburg-Eppendorf, Hamburg, Germany

**Keywords:** Pediatric acute lymphoblastic leukemia, INF-γ, TNF-α, Th1 cytokines

## Abstract

Immune milieus play an important role in various types of cancer. The present study focuses on the effect of Th1 cytokines on pediatric acute lymphoblastic leukemia (ALL). The reaction of ALL cell lines and patient-derived xenografts (PDX) to the most important Th1 cytokines TNF-α (tumor necrosis factor alpha) and IFN-γ (interferon gamma) is analyzed and correlated with the respective cytokine receptors and the intracellular signaling molecules. ALL cell lines and ALL PDX display a great heterogeneity in cell death after incubation with TNF-α and IFN-γ. Several samples show a dose-dependent and additive induction of cell death by both cytokines; others do not react at all or even display an increased viability. Apoptosis is the main type of cell death induced by Th1 cytokines in ALL cells. Over all leukemia cells analyzed, IFN-γ receptor (IFNGR) shows a higher expression than both TNF-receptors, resulting in higher phosphorylation of STAT1 (signal transducer and activator of transcription) compared to phosphorylation of NF-κB (nuclear factor kappa-light-chain-enhancer of activated B-cells) in the TNF pathway. The activation of STAT1 correlates with the amount of cell death after stimulation with Th1 cytokines. TNF-α and IFN-γ lead to heterogeneous reactions in ALL cell lines and ALL PDX but are able to induce cell death by apoptosis in the majority of ALL blasts. The correlation of a high expression of IFNGR and following activation of STAT1 with cell death indicates an important role for IFN-γ signaling in this setting.

## Introduction

There is evidence that immune mediators are associated with outcome in pediatric leukemia [1–3]. IL-10 (interleukin-10) and TNF-α as well as TGF-β (transforming growth factor β) and IFN-γ are assumed to have contrary effects in anti-leukemia immunity. Low production of the pro-inflammatory Th1 cytokines TNF-α and IFN-γ at diagnosis is associated with high-risk criteria and T-ALL**.** A high initial peripheral blast count correlates negatively with IFN-γ expression. These findings imply suppression of host Th1 immunity caused by leukemia [3]. Gene polymorphisms in the anti-inflammatory cytokines IL-10 and TGF-β genes, but not in TNF-α and IFN-γ genes, have an impact on allele frequency, risk group and prognosis in ALL [3].

TNF-α is considered to be a major pro-inflammatory mediator, with an optional capacity to induce apoptosis [4]. The paradoxical effect of TNF-α seems to be dose-dependent. Permanently produced low-dose TNF-α enhances tumor formation, growth, metastasizing and cachexia, while supra-physiological dosages of TNF-α can destroy tumor vascularity and induce necrosis and apoptosis in tumor cells [4, 5].

In non-Hodgkin’s lymphoma, acute and chronic myeloblastic leukemia and chronic lymphocytic leukemia high levels of TNF-α and its receptors are associated with poor outcome and therapy resistance [6, 7].

In ALL, TNF-α has been reported to exert either proliferative or cytotoxic effects on primary blast cells ex vivo [8]. TNFR-1 mainly induces apoptotic signaling by engaging a death domain (e.g., in bacterial response), whereas TNFR-2 predominantly regulates survival signaling (e.g., in immune cell activation) [9]. But TNFR-2 can also lead to induction of cell death by indirect mechanisms such as increasing the production of endogenous TNF-α [10].

Comparing the plasma levels of TNF-α of children suffering from B-ALL at diagnosis with healthy controls, higher levels were detected in ALL patients. Furthermore, a positive correlation between TNF-α levels with blast cell and white blood cell count was observed. The TNF-α levels in ALL patients were negatively correlated with S-phase leukemic cells and apoptotic cells but not with treatment response or survival [11].

Analyzing the role of the TNFRs in leukemia, a retrospective analysis of serum concentration of soluble TNF receptors (sTNFR-1 and sTNFR-2) in adult patients showed significantly elevated levels in AML (acute myeloblastic leukemia) and ALL. Moreover, in AML patients, a negative correlation between the sTNFR-1 levels and disease-free survival and overall survival was observed. In ALL, no such correlation was detected [12].

Alongside TNF-α, IFN-γ is the second major proinflammatory Th1 cytokine [13]. Genotyping in pediatric B-ALL patients revealed that IFN-γ high-expressing genotypes were present in patients of an older age at the time of clinical manifestation of leukemia. It is speculated that IFN-γ might suppress the outgrowth of malignant clones. In contrast, IFN-γ low-expressing genotypes were significantly more common in high-risk B-ALL patients. The association of polymorphic IFN-γ alleles with age at clinical presentation and risk group evokes a role for IFN-γ in immunosurveillance [14]. The immunomodulatory role of IFN-γ in B-ALL is also reflected by the analysis of ALL natural killer (NK) cells. At ALL diagnosis, the NK cells showed an inhibitory phenotype (downregulation of activating receptors and upregulation of inhibitory receptors) with impaired IFN-γ production and cytotoxicity [15].

Similar to TNF-α, both pro-tumorigenic and pro-apoptotic properties of IFN-γ have been described [13].

Senescence, a permanent arrest in G1/G0, is an important endogenous defense mechanism against an early stage of neoplasms in various tissues such as oncogene-induced senescence in lymphoma development [16]. The induction of senescence by IFN-γ and TNF-α has been described in several cancer forms such as breast cancer, rhabdomyosarcoma, and primary melanoma or sarcoma [17]. Recently, the induction of senescence has also been shown in AML cells by secretion of Th1 cytokines through tumor-specific T-cells [18].

Novel immune-therapeutics used against ALL have become an important pillar of therapy. Bispecific antibodies or CAR-T-cells (chimeric antigen receptor T-cells) can lead to release of large amounts of pro-inflammatory cytokines, with levels of TNF-α rising tenfold and levels of IFN-γ rising up to 1000-fold [19–21]. The direct effects of the released cytokines on the leukemia cells remain to be elucidated but IFN-γ definitely seems to contribute to the efficiency of cancer immunotherapy with immune checkpoint inhibitors in solid tumors [22, 23].

In the present study, we describe the reaction of ALL cell lines and ALL PDX to the Th1 cytokines TNF-α and IFN-γ and analyze the expression of the respective cytokine receptors and the main intracellular signaling pathway.

## Material and methods

### ALL cell lines

MHH-CALL-4, Nalm-6, Nalm-16, Reh, RS4;11 and KOPN-8 were purchased from the German Collection of Microorganisms and Cell Cultures GmbH (DSMZ) and cultured according to instructions.

### PDX

NOD.Cg-*Prkdcscid IL2rgtmWjl*/Sz (NSG) mice were purchased at The Jackson Laboratory and maintained under specified pathogen-free conditions in the research animal facility of the University of Tuebingen, Germany. All experimental animal procedures were conducted according to German federal and state regulations (local ethics approval number 448/2014BO1). Patient-specific leukemia was induced in NSG mice as described before [24]. The study was approved by the local ethics committee and written informed consent was obtained from the parents, in accordance with the Declaration of Helsinki. Upon engraftment, mice were euthanized, and bone marrow (BM) or spleen specimens of leukemia-bearing mice were analyzed immediately or stored at − 80 °C for de novo generation of patient-specific leukemia at later time points.

Patient characteristics of the samples used to generate the patient-derived xenografts (PDX) are displayed in Table 1.

**Table 1:** characteristics of patient-derived xenografts

PDX	Status of disease	Gender	Age (years)	Molecular genetics	Outcome
PDX_1	2nd relapse	Male	4.9	MLL-AF9	Death of relapse
PDX_2	Initial diagnosis	Female	2.1	–	Remission
PDX_3	Refractory disease	Male	4.5	–	Death of relapse
PDX_4	Initial diagnosis	Female	2.4	–	Remission
PDX_5	1st relapse	Male	3.1	atyp. TEL-AML1	Death of relapse
PDX_6	Initial diagnosis	Female	3.1	–	Remission
PDX_7	Initial diagnosis	Male	15.3	–	Remission
PDX_8	Initial diagnosis	Female	6.2	PBX1-TCF3	Remission
PDX_9	Initial diagnosis	Male	5.3	TEL-AML1	Remission
PDX_10	Initial diagnosis	Male	14.8	–	Remission
PDX_11	2nd relapse	Female	4.3	–	Death of relapse
PDX_12	Initial diagnosis	Male	4.0	TEL-AML1	Remission
PDX_13	Initial diagnosis	Female	4.4	TEL-AML1	Remission
PDX_14	3rd relapse	Male	14.8	–	Remission
PDX_15	Initial diagnosis	Female	13.9	PBX1-TCF3	Remission
PDX_16	Initial diagnosis	Male	6.0	–	Remission

### Flow cytometry

Flow cytometry was performed on an LSRII by BD Biosciences. Viability of cells was assessed in the forward scatter versus sideward scatter (FSC/SSC) plot. The percentage of specific cell death was determined using the following formula: specific cell death = (cytokine induced cell death − spontaneous cell death)/(100 − spontaneous cell death) × 100%.

### Annexin V/7-AAD assay

We used the FITC Annexin V Apoptosis Detection Kit with 7-AAD by Biolegend (Cat. 640922) according to manufacturer’s instructions. Staurosporine was used as a positive control.

### Caspase assay

The Apo-ONE® Homogeneous Caspase-3/7 Assay by Promega (#G7790) was used according to the manufacturer’s instructions. Staurosporine was used as a positive control.

### Cytokine receptors/ABC

The antibodies detecting TNF-α receptors were purchased from R&D Systems: TNFR-1 (CD120a)-APC (FAB225A), TNFR-2 (CD120b)-APC (FAB226A) and IFNGR (CD119)-PE were purchased from BioLegend (Cat. Nr. 308704).

The antigen binding capacity of the cytokine receptors was assessed by flow cytometry using Quantum simply cellular by Bangs Laboratories α-mouse IgG (Cat. 815).

### Cell cycle assay

For cell cycle analysis, we used the BrdU Flow Kit Cat. No. 559619 by BD Biosciences according to the manufacturer’s instructions. Incubation time with BrdU was chosen according to the doubling time of the cell line (1 h for most cell lines, 18 h for MHH-CALL-4).

### Phosphoprotein signaling 

The antibodies against the phosphorylated downstream signaling molecules were purchased from BD Biosciences: pNF-kBp65-PE-Cy7 (56035) and pSTAT1-BV421 (562985) as were the buffer solutions. All were used according to the manufacturer’s instructions.

### Statistics

Data were analyzed using Microsoft® Excel, Version 16.12 and IBM® SPSS Statistics Version 22 for Windows and GraphPad Prism9. Differences between subgroups were described by two-way ANOVA; correlation analysis was performed using the Spearman's nonparametric measure of rank correlation. Results with a *p* value of < 0.05 were considered statistically significant.

## Results

### Cell cycle analysis

**Fig. 1:**
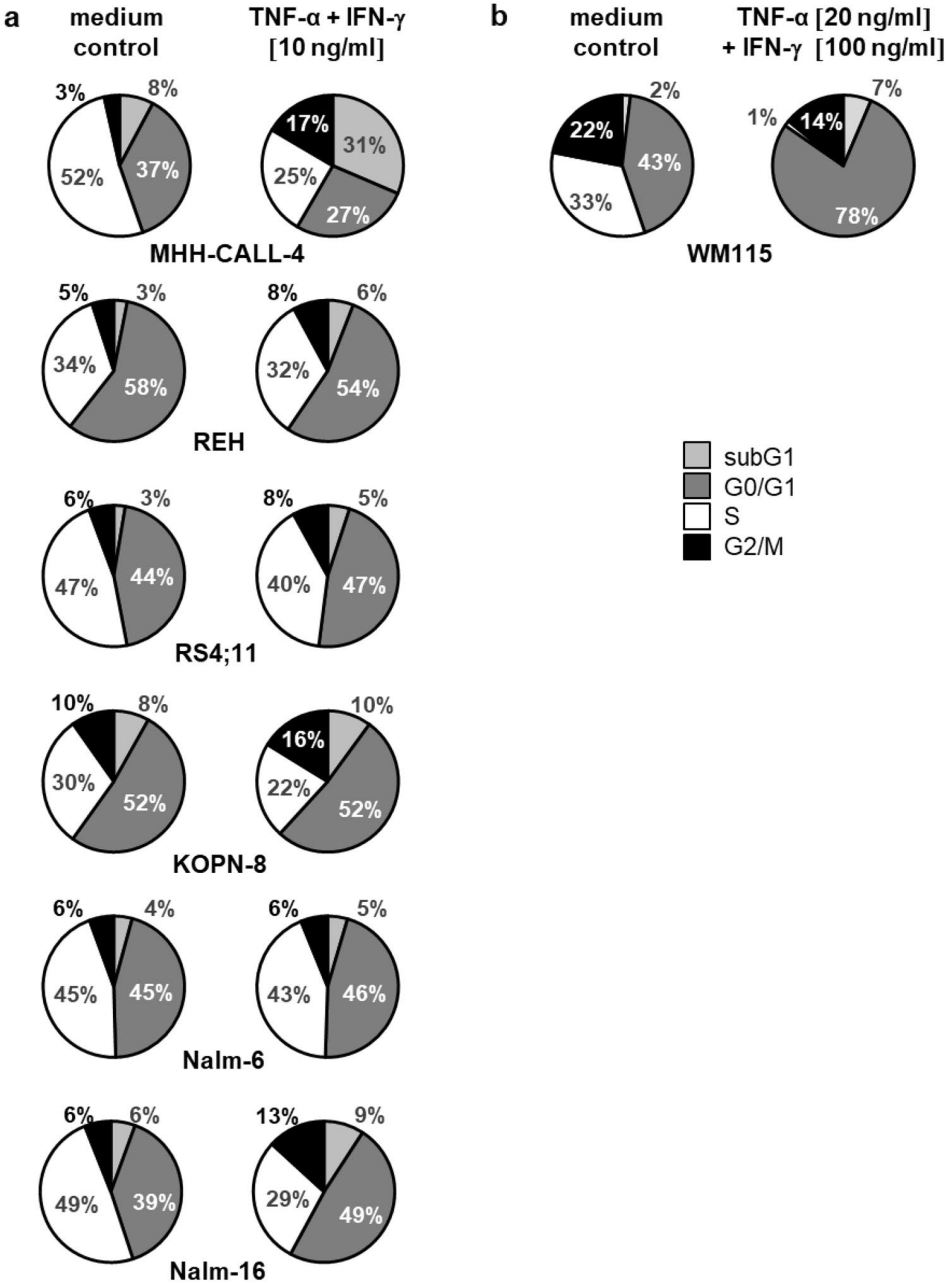
**a** Cell cycle changes in BCP-ALL cell lines after incubation with Th1 cytokines, **b** positive control melanoma cell line WM115 undergoing senescence after incubation with TNF-α and IFN-γ

In MHH-CALL-4 the incubation with TNF-α and IFN-γ leads to a decrease in cells in S-phase and a simultaneous increase in cells in subG1. Most other ALL cell lines show little or no changes in cell cycle after incubation with Th1 cytokines (slight increase of G0/G1 in RS4;11 and Nalm-16). The positive control, a melanoma cell line, goes into senescence with a strong reduction of S-phase and high increase in resting cells, as shown previously by Braumüller and colleagues [17] (Fig. 1).

### Specific cell death

#### B-cell precursor ALL cell lines

**Fig. 2:**
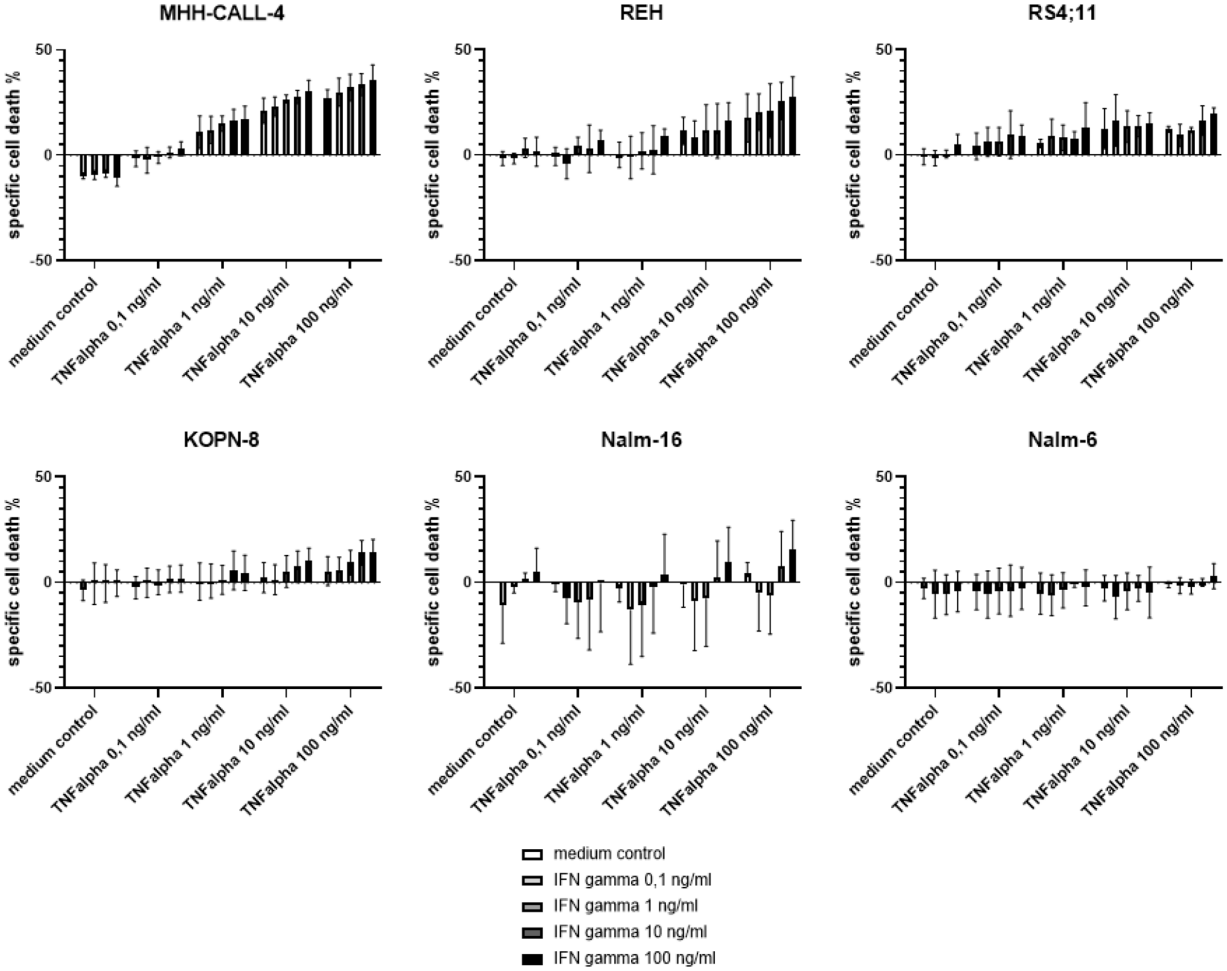
shows specific cell death of ALL cell lines by TNF-α and IFN-γ at increasing concentrations after an incubation time of 72 h; MHH-CALL-4, REH, RS4; 11 and KOPN-8 show significant reactions to both cytokines in a two-way ANOVA analysis, Nalm-16 only shows a discrete reaction to IFN-γ and Nalm-6 does not show any significant change in cell viability under exposure with Th1 cytokines

The induction of cell death in six B-cell precursor ALL cell lines (MHH-CALL-4, NALM-6, REH, RS4;11, NALM-16, KOPN-8) incubated with different concentrations of TNF-α (0.1 ng/ml,1 ng/ml, 10 ng/ml, 100 ng/ml) and IFN-γ (0.1 ng/ml, 1 ng/ml, 10 ng/ml, 100 ng/ml) was examined alone and in combination at multiple points in time (24 h, 48 h, 72 h). Four cell lines show a dose-dependent induction of cell death by both cytokines, the highest amount of cell death induction detected in MHH-CALL-4. The two cell lines Nalm-6 and Nalm-16, however, did not undergo cell death under incubation with Th1 cytokines. Nalm-16 even showed a slight tendency toward increased viability under certain concentrations (Fig. 2).

#### ALL patient-derived xenografts (ALL PDX)

**Fig. 3:**
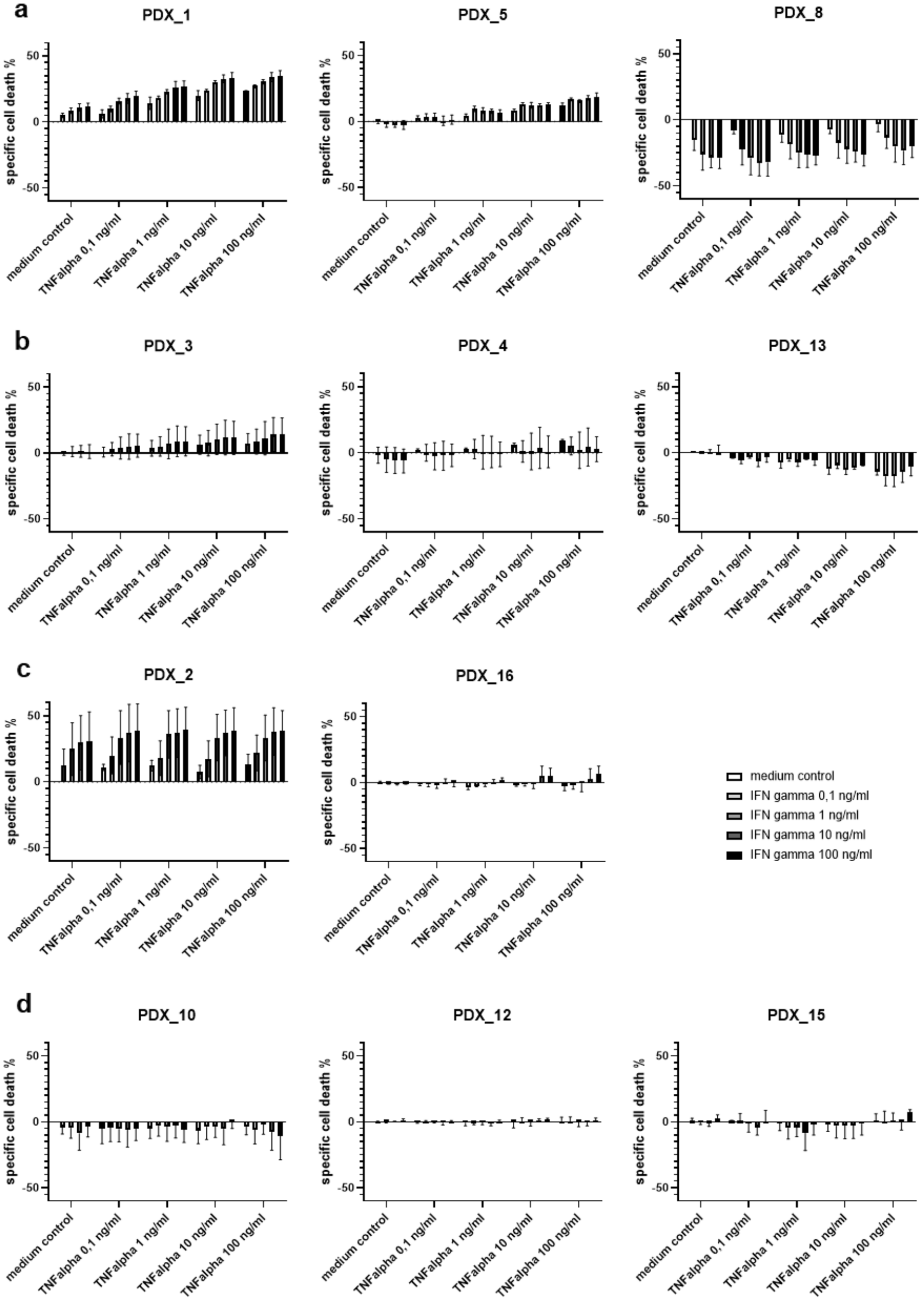
shows specific cell death of patient-derived xenografts (PDX) by TNF-α and IFN-γ at increasing concentrations after an incubation time of 48 h, **a** shows three representative PDX out of seven with a significant change in cell viability due to both TNF-α and IFN-γ, **b** shows three PDX out of four with significant changes only to TNF-α, **c** displays the two PDX with significant changes only to IFN-γ and **d** displays the three PDX without viability changes under exposure of Th1 cytokines (significance according to a two-way ANOVA analysis)

The same TNF-α and IFN-γ concentrations as noted above for the cell lines were used to analyze specific cell death induction in ALL PDX. The PDX cells showed even greater inter-individual variance of responses to exposure with Th1 cytokines. In seven PDX samples, a significant change in viability by TNF-α and IFN-γ was observed, albeit some PDX displaying an increase in cell death whereas others showed a better viability under Th1 cytokine stimulation (Fig. 3a). Other PDX shown in Fig. 3b, c only showed a significant reaction to one of the cytokines and three PDX did not show any measurable change in cell viability under Th1 cytokine stimulation (Fig. 3d). Also, the level of specific cell death or increased viability was markedly different between the different PDX.

### Induction of apoptosis

Several cell lines and PDX displayed a substantial increase in specific cell death. We therefore performed further assays to determine the level of apoptosis induced by Th1 cytokines. We used concentrations of 10 ng/ml for TNF-α and IFN-γ.

We stained annexin V and 7-AAD and additionally assessed caspase-activation by cleavage of effector caspases 3 and 7 for two cell lines and all the ALL PDX.

**Fig. 4:**
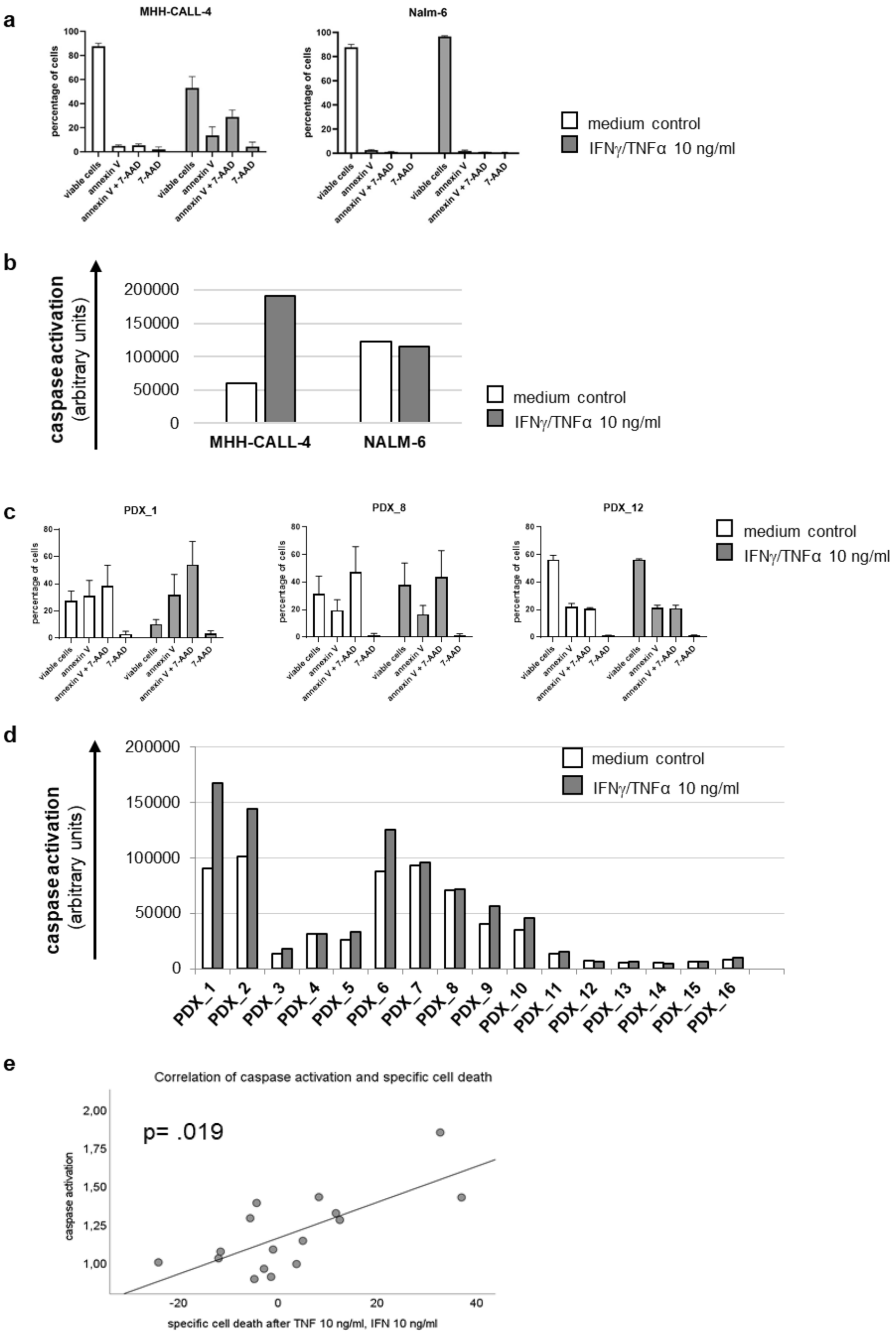
shows apoptosis after incubation with Th1 cytokines in two ALL cell lines and in ALL PDX; **a** displays levels of annexin V and 7-AAD in the cell lines with the most divergent reaction to TNF-α and IFN-γ MHH-CALL-4 and Nalm-6, **b** shows the amount of effector caspase activation in MHH-CALL-4 and Nalm-6, **c** displays three examples of ALL PDX one with increase in annexin V after Th1 cytokines and two without, **d** shows the caspase activation levels of all ALL PDX analyzed and **e** displays the significant correlation between the amount of specific cell death and the activation of effector caspases in ALL PDX after incubation with TNF-α and IFN-γ

The ALL cell line MHH-CALL-4 shows a marked increase of annexin V positive cells as well as of caspases 3 and 7 after incubation with Th1 cytokines, whereas Nalm-6 did not undergo apoptosis after treatment with IFN-γ and TNF-α (Fig. 4a, b). These results mirror their reaction in the assessment of specific cell death (Fig. 2) where MHH-CALL-4 showed a marked increase in specific cell death but Nalm-6 stayed inert to various concentrations of Th1 cytokines. Not surprisingly, in the ALL cell lines, the percentage of annexin V positive cells after treatment with Th1 cytokines correlates positively with percentage of cells in subG1 and negatively with percentage of cells in S-phase. There is also a positive correlation between percentage of annexin positive cells and percentage of cells in G2/M-phase.

The PDX showed varied reactions in apoptosis induction, as expected by their differential amount of specific cell death after incubation with IFN-γ and TNF-α. PDX with a high level of specific cell death also showed higher levels of annexin V positive cells and stronger activation of caspases 3 and 7 (Fig. 4c, d). There is a significant positive correlation between the amount of specific cell death and the level of caspase activation in ALL PDX exposed to Th1 cytokines (Fig. 4e).

### Quantification of cytokine receptors

**Fig. 5:**
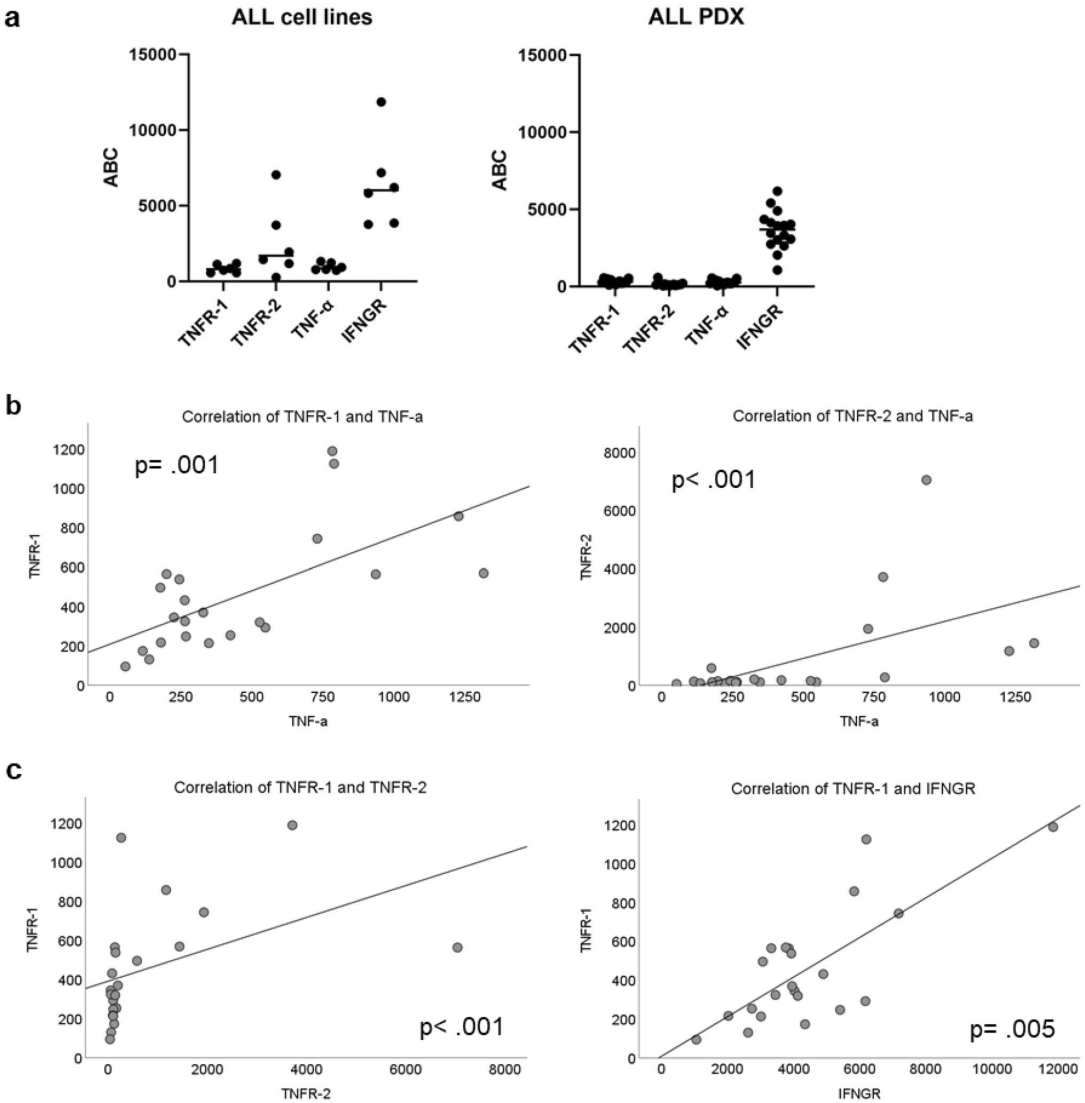
**a** shows the antigen binding capacity (ABC) for TNFR-1, TNFR-2, TNF-α and IFNGR in ALL cell lines and ALL PDX; **b** shows the significant correlation between TNF-α and both TNR-receptors, and **c** displays the correlation between TNFR-1 and both TNFR-2 and IFNGR

We analyzed the expression of receptors for IFN-γ and TNF-α on the surface of leukemia cells by quantifying the antigen binding capacity for TNF-α receptor type 1 (TNFR-1), TNF-α receptor type 2 (TNFR-2), IFN-γ receptor (INFGR) and membrane bound TNF-α. The expression of TNF receptors and TNF-α is expressed at relatively low levels, especially in the PDX samples. The ALL cell lines display a higher level of all three receptors analyzed. IFNGR is generally expressed at a much higher level with large intra-individual differences and with a less clear separation between cell lines and PDX (Fig. 5a). The expression of both TNF-receptors correlates with the level of membrane bound TNF-α (Fig. 5b), TNFR-1 expression also strongly correlates with the expression level of TNFR-2 and IFNGR.

### Signaling transduction pathways

After assessing the main cytokine receptors for TNF-α and IFN-γ we analyzed the intracellular signal transduction via phosphorylation of the main downstream effectors of both cytokines. For TNF-α, we focused on the phosphorylation of NF-κB (nuclear factor kappa-light-chain-enhancer of activated B-cells) and for IFN-γ on phosphorylation of STAT1 (signal transducer and activator of transcription). The activation of the signaling transduction pathways by TNF-α and IFN-γ was quantified by comparing the phosphorylation state of the transcription factor in medium control with the phosphorylation state after incubation with the respective cytokine.

**Fig. 6:**
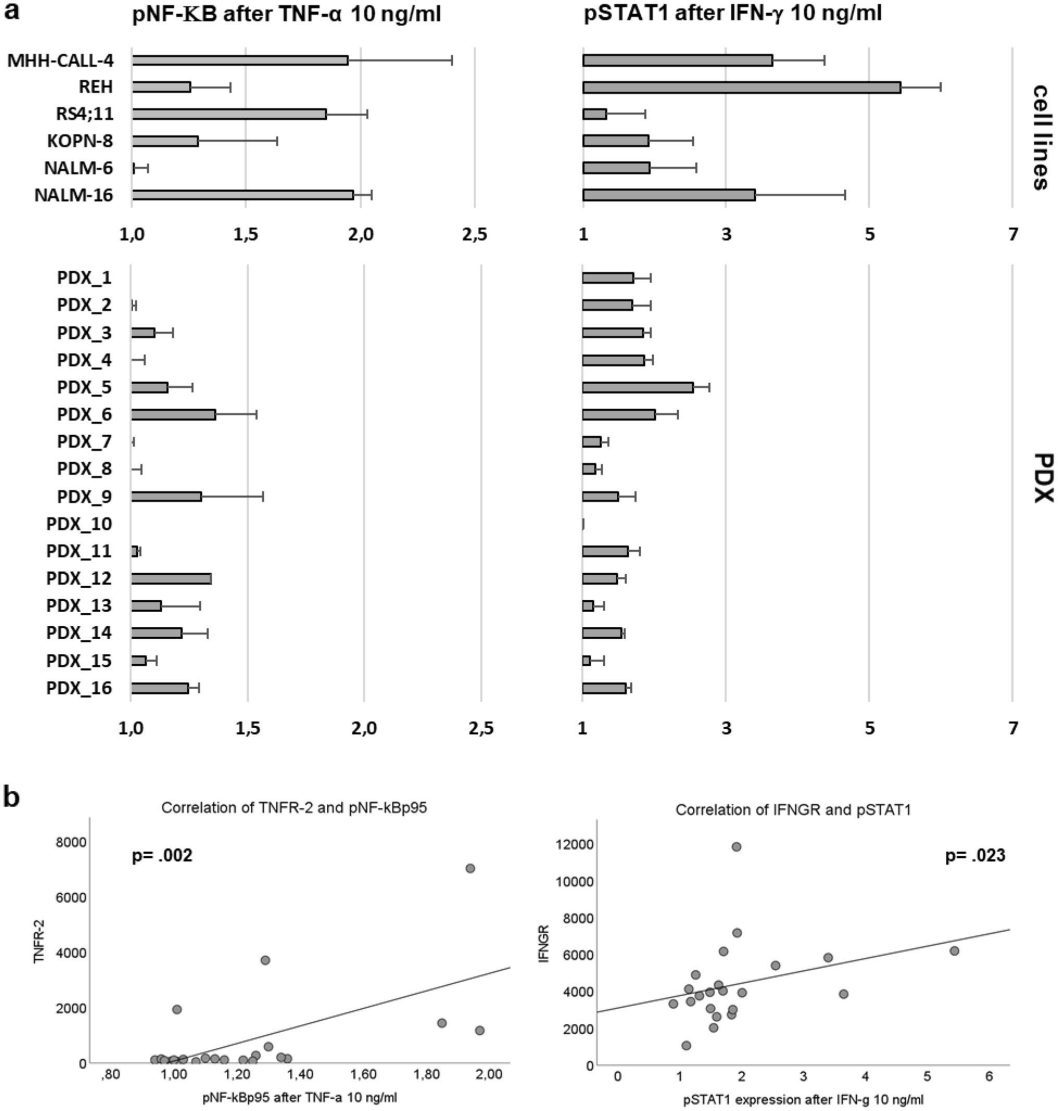
**a** shows the level of phosphorylation of NF-κB and STAT1 after stimulation with TNF-α and IFN-γ, respectively, **b** shows the correlation between the amount of NF-κB phosphorylation and the expression of TNFR-2 (left) and the correlation between the amount of STAT1 phosphorylation and the expression of IFNGR (right)

**Fig. 7:**
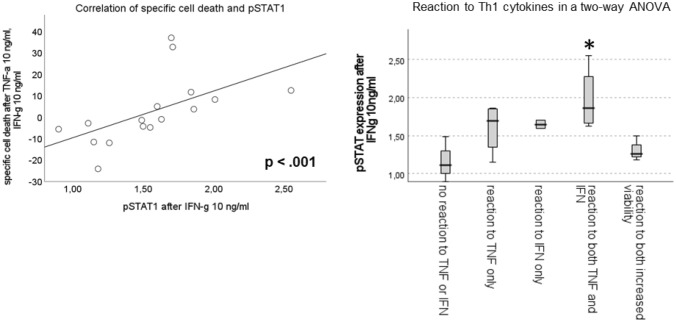
shows the correlation between the activation of STAT1 and the amount of cell death after stimulation with Th1 cytokines (left) and the amount of pSTAT1 according to the reaction to Th1 cytokines (right)

The phosphorylation level of NF-κB after incubation with TNF-α was extremely diverse and varied largely between cell lines and PDX, some samples displaying a strong phosphorylation, while others did not show any increase in phosphorylation in the presence of TNF-α compared with medium control (Fig. 6a). The expression level of TNFR-2 correlates with phosphorylation of NF-κB (Fig. 6b). The increase in phosphorylation of STAT1 after incubation with IFN-γ was higher in general and present in almost all samples. For pSTAT1, there is also considerable variability between the different ALL samples (Fig. 6a). The expression level of IFNGR significantly correlates with the activation of STAT1 over cell lines and PDX and the phosphorylation of STAT1 strongly correlates with a higher amount of cell death after treatment with Th1 cytokines. The phosphorylation of STAT1 is highest in the PDX with decreased viability after being exposed to Th1 cytokines, especially in the samples which showed significant induction of cell death after incubation with both cytokines (Fig. 7).

## Discussion

The reactions of ALL cell lines and PDX to incubation with the pro-inflammatory cytokines TNF-α and IFN-γ were highly variable, as was the expression of the cytokine receptors. In our analysis, the amount of TNF receptors positively correlate with each other. ALL cells with high amounts of TNFR-1 also have higher amounts of TNFR-2 and of TNF-α.

TNFR-1 is a typical death receptor triggering apoptotic and necroptotic signaling, whereas TNFR-2 lacks a death domain. TNFR-2 induces NF-κB signaling via several pathways, can attenuate TNFR-1-induced classical NF-κB signaling and sensitize cells for TNFR-1-induced cell death. But TNFR-2 can also activate the alternative and classical NF-κB pathway which upregulates anti-apoptotic proteins and proliferation promoting factors [25]. The survival-promoting effect of endogenous TNF-α by TNFR-2 and PI3K/protein kinase B signaling has been demonstrated in ALL cells [26].

We see a significant positive correlation between the expression of TNFR-2 and the activation of NF-κB as well as a negative correlation between the antigen binding capacity of TNFR-2 and the level of annexin V positive cells after incubation with Th1 cytokines.

Despite a clear positive correlation between the expression of TNFR-1 and TNFR-2 with membrane bound TNF-α, we see no association between the antigen binding capacity of either TNF receptor or the membrane bound ligand TNF-α with the induction of cell death after incubation with Th1 cytokines.

The IFN-γ receptor showed a high antigen binding capacity on all ALL samples, a finding which has previously been shown in AML [27]. Similar to our results, IFN-γ affected cell proliferation and regulation of apoptosis in AML cells and induced reproducible divergent effects on the percentage of viable cells after 48 h of culture with an increased viability in some and a decreased viability in others [27].

The level of cell death after incubation with Th1 cytokines in pediatric ALL strongly correlates with the expression of IFN-γ receptor and subsequently also the phosphorylation of STAT1 in pediatric ALL (cell lines as well as PDX). Our results indicate that in pediatric ALL the IFN-γ signaling pathway plays a major role in the cell death induction after Th1 cytokine exposure.

TNF-α and IFN-γ have been shown to synergistically induce cell death in different cell types such as murine hepatoma cells [28], a myeloblastic leukemia cell line [29] and oligodendroglioma cells [30]. The exact mechanism of the synergistic cytotoxicity is unknown.

But the induction of senescence by TNF-α and IFN-γ in several solid tumors may be part of the explanation [17]. Th1 cytokines secreted by tumor-specific T-cells have very recently been shown to induce senescence in AML cells [18]. The cell cycle changes shown here in BCP-ALL cell lines after incubation with TNF-α and IFN-γ were only minor and did not reflect a senescent phenotype but rather apoptosis induction in some cell lines.

Many ALL samples showed an additive or even synergistic effect of both Th1 cytokines on specific cell death, but others showed no increase in cell death at all. The expression of IFN-γ receptor and the consecutive activation of STAT1 were the main reason identified in this work but is surely not the only answer to the differential reaction of ALL cells to exposure to TNF-α and IFN-γ.

As mentioned in the introduction, several studies have investigated germline polymorphisms in cytokine genes such as the association of TNF and IL-10 genotypes with adverse outcomes in solid and lymphoid malignancies [2] or gene polymorphisms in IL-10 and TGF-β genes having an impact on allele frequency, risk group and prognosis in ALL [3]. But only few have focused on the measurement of plasma levels of the immune mediators. In Hodgkin’s lymphoma, the levels of TNF-α and its soluble receptors at diagnosis correlate with clinical features and outcome [31].

Individual oncological therapies are available including TNF-α and IFN-γ. The combination of TNF-α, IFN-γ and melphalan in an isolated limb perfusion setting shows impressive remission rates in irresectable soft tissue sarcomas [32] and melanoma [33] and remains a possible treatment option for advanced disease [34, 35]. A positive effect on survival rates of patients suffering from bladder carcinoma was demonstrated for IFN-γ given intravesically [36]. There were also encouraging results for the intraperitoneal treatment in ovarian cancer [37]. In contrast to these local treatments, the systemic administration of recombinant human IFN-γ in patients with acute myeloblastic leukemia and myelodysplastic syndromes did not lead to hematological responses and was only poorly tolerated [38]. In small-cell lung cancer IFN-γ sensitized normal lung tissue for the effects of irradiation but induced severe side effects [39]. Clinical studies of IFN-γ against colon cancer or metastatic renal carcinoma also found no clear benefit [40, 41].

Apart from the local therapeutic possibilities in solid tumors mentioned above, the systemic application of exogenous TNF-α and IFN-γ does therefore not constitute a realistic anti-tumor therapy in leukemia. Many novel immunotherapeutic agents, however, lead to an increase in endogenous Th1 cytokines.

The T cell engaging CD19/CD3-bispecific antibody blinatumomab has become a standard treatment in relapsed ALL. After the first infusion a transient release of cytokines including IFN-γ and TNF-α can be observed [42]. In an in vitro chronic lymphocytic leukemia (CLL) study, culture with blinatumomab significantly increased the levels of IFN-γ, TNF-α, TNF-β and IL-8 (interleukin-8) [43].

Another potent immunotherapeutic option for relapsed or refractory ALL patients is CD19-targeted CAR (chimeric antigen receptor)-T-cell therapy. After infusion, a massive expansion of CAR T-cells leads to secretion of vast amounts of IFN-γ and TNF-α and further stimulates bystander immune cells and endothelial cells [44]. Cytokine levels were shown to peak five to ten days after infusion and reach concentrations up to 1000-times the value of baseline, especially for IFN-γ [21]. These cytokine release syndromes are not yet fully understood and it is not clear whether the high levels of cytokines have a direct anti-leukemic effect themselves.

For immune checkpoint inhibition, it has been shown that gene expression profiles from tumor tissue samples of different solid tumors who responded to anti-PD-1 therapy had higher expression of IFN-γ-related genes [22]. In mouse models, the clinical benefit of cancer immunotherapy is reduced in mice bearing INFGR^−/−^ tumors [45].

In our analysis, the expression of IFNGR and phosphorylation of STAT1 was clearly correlated with specific cell death of ALL samples after incubation with Th1 cytokines. The expression level of IFNGR might be a prediction marker for the clinical response to immunotherapeutic approaches in ALL, similar to the results described above for solid tumors.

The limitation of our study is the mere in vitro character and the moderate number of individual leukemia samples analyzed. Statistical significance also does not necessarily reflect causal relationships. This work nevertheless demonstrates that the Th1 cytokines TNF-α, and IFN-γ can lead to apoptosis in BCP-ALL cells, especially IFN-γ and its receptor seem paramount for the induction of cell death in this setting. The strong heterogeneity of reaction between different ALL cells to the Th1 cytokines warrants further investigation and might lead to future tailored immunotherapeutic concepts.

## Data Availability

The datasets generated during and/or analyzed in the current study are available from the corresponding author on reasonable request.
